# A Dependable Localization Algorithm for Survivable Belt-Type Sensor Networks

**DOI:** 10.3390/s17122767

**Published:** 2017-11-29

**Authors:** Mingqiang Zhu, Fei Song, Lei Xu, Jung Taek Seo, Ilsun You

**Affiliations:** 1 School of Electronic and Information Engineering, Beijing Jiaotong University, Beijing 100044, China; mqzhu@bjtu.edu.cn (M.Z.); fsong@bjtu.edu.cn (F.S.); 2 Graduate School, University of International Business and Economics, Beijing 100029, China; xulei@uibe.edu.cn; 3 Department of Information Security Engineering, Soonchunhyang University, Asan-si 31538, Korea; seojt@sch.ac.kr

**Keywords:** belt-type sensor networks, security, the hop distance, energy-efficient, connectivity

## Abstract

As the key element, sensor networks are widely investigated by the Internet of Things (IoT) community. When massive numbers of devices are well connected, malicious attackers may deliberately propagate fake position information to confuse the ordinary users and lower the network survivability in belt-type situation. However, most existing positioning solutions only focus on the algorithm accuracy and do not consider any security aspects. In this paper, we propose a comprehensive scheme for node localization protection, which aims to improve the energy-efficient, reliability and accuracy. To handle the unbalanced resource consumption, a node deployment mechanism is presented to satisfy the energy balancing strategy in resource-constrained scenarios. According to cooperation localization theory and network connection property, the parameter estimation model is established. To achieve reliable estimations and eliminate large errors, an improved localization algorithm is created based on modified average hop distances. In order to further improve the algorithms, the node positioning accuracy is enhanced by using the steepest descent method. The experimental simulations illustrate the performance of new scheme can meet the previous targets. The results also demonstrate that it improves the belt-type sensor networks’ survivability, in terms of anti-interference, network energy saving, etc.

## 1. Introduction

Security has become a major challenge for Internet of Things (IoT) research. It cannot be ignored in massive IoT applications as well. With the rapid expansion of the IoT, a variety of different wireless communication technologies and network structures are constantly integrating, including Wireless Sensor Networks (WSNs), RFID, mobile vehicular networks, mobile networks, 4G communication networks, WiMAX and cable broadband, etc. Meanwhile, the network communication environment is becoming more and more complicated. Compared with existing wireless networks, security issues such as reliable relationships between entities and positioning will be more difficult in the IoT [1]. These problems cannot be solved simply through existing network security solutions. In various objects and all kinds of network communication scenarios, how to ensure the reliability of information resources, the stability of information transmission and the security of information space have become important and urgent problems.

As an important component of the IoT, belt-type sensor networks are widely used in ribbon-like monitoring areas such as rivers, highways, tracks, bridges, pipelines and the industry fields. It is indeed a special IoT branch that can provide information exchange between items in a ribbon area. Due to the terrain and surrounding environments, the topology of the belt-type sensor network presents a narrow, elongated strip as a whole. In this type of network, a large number of nodes with limited resources are often deployed in one-dimensional or approximate one-dimensional linear spaces. Information transmission can only be carried out along a few paths, with a certain direction. The communication range of each node in the sensor networks cannot be covered by the gateway node. Therefore, the node which is far away from the target needs to transmit data in a multi-hop way. More seriously, belt-type sensor networks have an open nature which is particularly vulnerable to all kinds of attacks. When it comes to widespread applications, security issues cannot be ignored.

Location is a crucial parameter in the belt-type sensor network. Normally, the data itself is meaningless if it contains no corresponding location information. In order to ensure effective interconnection among individuals within the network coverage, the data should be located. Especially when a sensor node detects an emergency, its location information should be quickly and accurately identifiable [1,2]. Node location information is critical to the effectiveness of sensor networks applications. As the core support of the IoT, secure location technology is receiving more and more attention. In such a situation, designing a dependable localization algorithm for survivable belt-type sensor networks is a realistic challenge. It is also a meaningful work to improve the next-generation wireless technologies for the IoT [3].

The security location algorithm based on the non-ranging method is a research hotspot, which usually use the estimated distances between nodes to calculate the position [4]. At present, there are more research results for sensor networks deployment strategy in open and flat environments [5,6,7,8,9,10,11]. For an ideal environment without malicious interference, most algorithms can achieve good positioning performance. However, as the technology is constantly updated and the application scenes become more complicated, it is clear that the existing algorithms cannot maintain the capacity, especially for the situation which requires a higher security level. In recent years, there have been many studies and reports on sensor network positioning [12,13,14,15,16,17,18,19,20]. Numerous non-ranging algorithms are only available for simple environments without complex disturbances. As a kind of classical non-ranging positioning algorithm, the Distance Vector Hop (DV-Hop) algorithm has been widely used and studied in depth for node location [1,5,21]. However, there are relatively few studies on algorithms which are suitable for special environments such as belt-type, crossover, and annular areas. For special occasions, the node deployment and positioning requirements are different compared with open flat space [22,23]. Generally, any kind of security algorithm will consume part of the available resources, including the computing energy and communication costs. Therefore, for a specific positioning system, we need to consider the system application background, security requirements of attack models, resource conditions and other factors [24,25]. Since special node deployments have different effects on network connectivity, many existing algorithms are greatly limited by their capacity [26,27,28,29,30,31,32].

The motivation of this paper is that the nodes cannot always achieve correct and stable positioning information, which may trigger network security risk. In particular, the wireless transmission medium is susceptible to temperature, humidity and other environmental impacts. Any failures or errors may generate unpredictable security risks. In this scenario, the node location method which is applicable for the wide area cannot be used directly in the long zone. At the same time, the resources of a single node are limited and it is difficult to add additional devices for positioning. In this paper, we fully consider the special structure of the region to deploy the belt-type sensor networks. More importantly, the addition of extra hardware devices is avoided and the network infrastructure is utilized as much as possible to locate the target. If the non-ranging method is used directly, the positioning accuracy cannot meet the requirements well. In order to get more precise results, an iterative refinement mathematical treatment method is introduced during the process. The limited resource of a single node is an important prerequisite, which has been fully considered. For the contradiction between energy consumption and positioning accuracy, an acceptable balance is established. We make the following contributions:

(1) We design a node deployment mechanism that could effectively save energy.
(2) A hop-distance calculation method that can eliminate blurring is proposed.
(3) We improve the accuracy of the proposed algorithm.

The remainder of our paper is organized as follows: Section 2 presents the modeling of the belt-type topology and node deployment mechanism based on energy-efficiency. Section 3 describes the improvement measures of the proposed algorithm in different stages from three aspects. How to determine the security of location information is also discussed in this section. Section 4 illustrates our simulations and experimental results. Finally, the conclusions are presented in Section 5.

## 2. Topological Modeling

In this section, we analyze the application environment of the algorithm, and model the belt-type sensor networks.

### 2.1. Model Requirements

**Figure 1:**
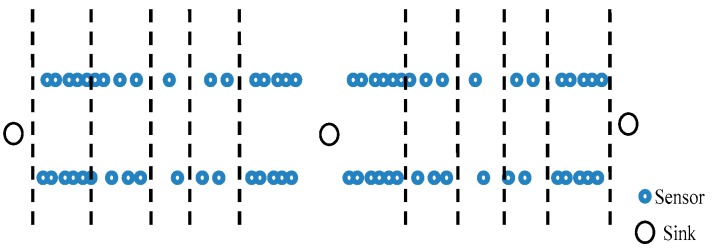
Adiagram of the network topology.

When deploying a wireless sensor network, if an aircraft is utilized to randomly distribute the anchor nodes, this may cause some nodes to be closely adjacent or overlapping, resulting in waste of resources and making many unknown nodes unable to be located. We use the packet structure model as the research basis, considering the characteristics of belt-type networks and the formation mechanism of the regional nodes when designing the topology. Grouping nodes in the network can improve the efficiency of communication channels and effectively control the resources occupied by the activity. The packet structure model is a common application object in sensor networks which is also the basis of an effective node deployment. The network topology is shown in Figure 1. In belt-type sensor networks, the normal nodes are usually placed along the edges of the coverage area, which makes it look like they are all in an extended line. For the sake of convenience, the nodes have the same information processing and communication capabilities in our model. Each type of node has its own sequence label (ID). The spatial distribution of nodes conforms to the following two principles: first, all nodes in the network are in the coverage of the whole detection area; secondly, in each sensor node group, the central node automatically becomes the group center, and the rest of the sensor nodes in each group can be covered by the central node of this group. The source node is a network node that acts as a sender to transmit the original packet. We name it “Source” for short. Sink, also known as “sink node”, is responsible for connecting the sensor network to other networks. Normally, it can be considered as a gateway. The node with relay function is named as the “relay node”. They are able to transmit data and information in a wireless, multi-hop manner. The beacon node is also known as an “anchor node” which belongs to a node that already knows its own position.

**Figure 2:**
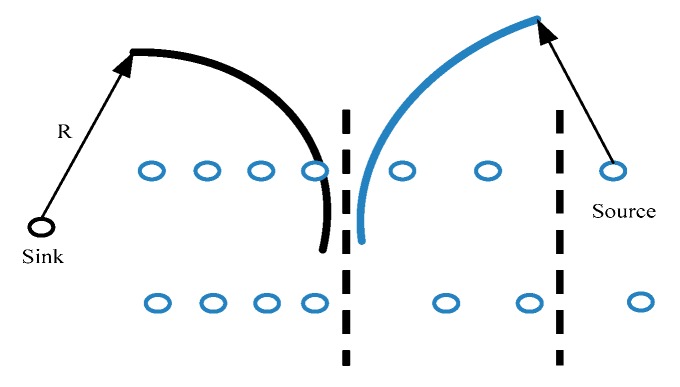
Node communication radius.

In belt-type sensor networks, the communication between a sensor node and a monitoring base station should be executed through a relay node in the multi-hop way. Therefore, the closer the node is with the monitoring station, the more data it needs to forward. After operating for a long time, the network can easily form a “hot zone” that increases the burden of the whole network. The energy consumption is increasing quickly as well [9,30]. Neighbor nodes near the base station are prone to “empty energy” phenomena. The emergence of the hot zone makes the number of nodes (near the base station) continuously accumulates, and the activity occupies a large amount of the communication bandwidth. The calculation ability of the node is severely reduced, which can cause the rate of packet loss to rise. Therefore, the location valid information cannot be effectively passed and tested, and the positioning accuracy is finally affected. The network of nodes loses communication and computing power, which can easily cause the network down and positioning failure. To study the problem more effectively, we assume that:

(1) The sensor nodes are distributed in a wide rectangular area (length is *L* and width is *M*, *L* >> *M*). The central gateway node is located in the middle of the strip area, and the sink node is located at the left side. The communication between the sensor node and the gateway node adopts the multi-hop mode, and the communication radius of the node is *R*. The distances between most sensor nodes and base stations are greater than the *R* of the nodes themselves. The nodes in the group can communicate directly with the sink nodes, and the communication distances between the intergroup nodes are one-hop. At this point, the sink node and the source node communication radius should be greater than the length of the group (shown in Figure 2).
(2) Each sensor node has a unique ID, and they can carry out information perception and collection independently. They can also send their own information through the wireless channel to the gateway node. In a unit area, let the rate of data generation is λ and let the initial energy of the sensor node is e.

### 2.2. Regional Energy Consumption

**Figure 3:**
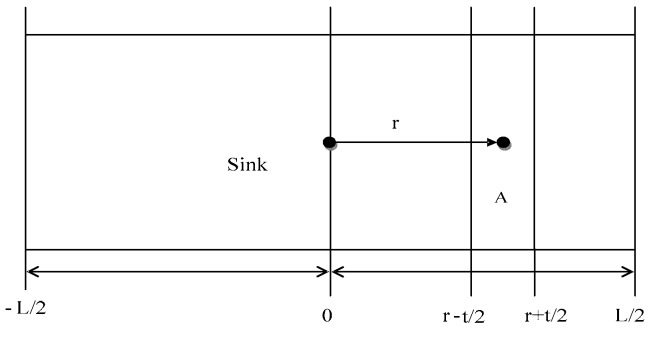
The network area diagram for analyzing energy consumption.

The process of exchanging information between distant nodes consumes a lot of energy in belt-type sensor networks. In a certain region, the life cycle of the sensor node can be approximately defined as the total energy of the region divided by the rate of energy consumption. To facilitate the analysis of energy consumption in the network area, we selected a small area which named as “A”. In order to maximize the extension of the life cycle of the sensor networks, the period of each sensor node should be set in a relatively close range. Therefore, the ratio of the energy consumption to the total energy in each region is set to a constant. According to the above analysis, the network horizontal range is set in [−L/2, +L/2], and the gateway nodes are arranged in the symmetrical position. The network area used to analyze energy consumption is shown in Figure 3.

Most of energy consumption in sensor networks mainly occurs during the data transmission. According to the classical signal propagation theory [9], the energy consumption of the transmitted information *E_T_*(*l*, *d*) can be expressed as Equation (1):

(1) $$E_{T}(l,d)=E_{e}(l)+E{\left(l,d\right)}_{\mathrm{amp}}$$

where l is the length of the packet, d is the transmission distance, $E_{e}(l)$ is the energy consumption of the circuit, $E{\left(l,d\right)}_{\mathrm{amp}}$ is the energy consumption parameter for the emitter. The energy consumption of the received information *E_R_*(*l*) can be expressed as Equation (2):

(2) $$E_{R}(l)=lE_{e}$$

From the Equations (1) and (2), we can achieve the total energy loss rate of the whole region. As shown in Equation (3).

(3) $$(E_{R}+E_{T})\int_{r+\frac{t}{2}}^{\frac{L}{2}}\lambda dx+E_{T}\int_{r-\frac{t}{2}}^{r+\frac{t}{2}}\lambda dx$$

The energy consumed by sending and receiving unit data are represented by *E_T_* and *E_R_* respectively. *t* is the communication distance of a single node. *r* stands for the distance between the reference node and the base station. When *r* is not equal to *t* and *r* >> *t*, the node density of the region (ρ(r)) is approximately $2n(L-2r)/L^{2}$. In summary, if the sensor node is closer to the gateway node, its distribution density should be larger. Because the node which is closer to the gateway node not only needs to send the collected perceptual data, but also to forward the information collected from others. Theoretically, more nodes need to join the network to share the communication consumption, achieve balanced load and extend network survival time.

### 2.3. Node Deployment Method

**Figure 4:**
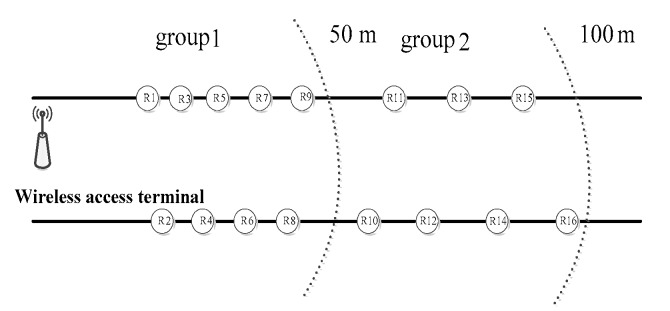
Nodes deployment of group routing protocol.

Let the total number of sensor nodes in the network be *N*. These nodes are subdivided into *n* groups in a belt-type zone. According to the physical topology environment and the control strategy on node density described in Section 2.1 and Section 2.2, the collection of each node is grouped and constructed. Both the effective communication radius of the node and the spacing among different groups are set to 50 m. At the same time, the group number of the node is assigned to each node. The group number of the sink node is 0, and the first group of nodes is with only one hop distance from the sink node. For the neighbor nodes, the distance between the other nodes and the sink node is gradually increased. For nodes at two-hops, three-hops and four-hops from the sink node, their group numbers are set to 2, 3 and 4, respectively. All the other groups can be numbered with similar method. In a range controlled by a gateway node, the source node is distributed from high to low on both sides according to the distribution density. Within the jurisdiction of a single gateway node, the distribution of each node is shown in Figure 4.

### 2.4. Node Activation Mechanism

In WSN, most member nodes should be dormant to save energy consumption. However, the nodes which are located at each group boundary or perform specific tasks should be in an operational state, i.e., The network does not need all nodes in work mode. If a reasonable sleep/wake mechanism is used, we can greatly reduce the network energy costs. Most sensor networks adopt IEEE 802.15.4 as the Media Access Control (MAC) layer protocol. It already contains a periodic sleep/wake mechanism. However, in the band topology, if the wake cycle of each node in the network is exactly the same, it will easily cause a packet internal node to generate large congestion when sending and receiving data information. As a result, the energy load of each node will generally increase. In order to adapt the network model based on energy-efficient strategy, the algorithm should use a sleep/wake-up scheduling mechanism based on a dynamic reconfiguration strategy as follows:

(1) When the target node enters the monitoring area, the sensor node starts searching and receiving the broadcast flood message “Request-MSG” issued by the target. According to the chronological order, sink node records the minimum hop number information “Hop-count” and its own identification information “Node-ID”. Subsequently, all information about anchor nodes in the neighbor area of the target node is stored and the initial positioning tree is constructed.
(2) Based on the network topology of the target node and neighbor anchor nodes in the sensing area, the two sets of nodes which need to wake up or keep the sleeping (low power state) are estimated and sorted. Once they are determined, the message “Wakeup-MSG” is immediately sent to the target, wake up and activate anchor nodes with *h* hop from the target node, launch them into working state and holding. After all above actions are completed, the message “Prune-MSG” is sent to the target, and the anchor nodes are cleared once again when the locating tree is created.
(3) According to the business type, we constantly and dynamically reconstruct the locating tree. As the target node moves and the sensing area continually changes, the anchor nodes that need to participate in the location continue to wake up or to remain dormant.

## 3. Hop-Distance Estimation Correction

In this section, we examine the connectivity among nodes and correct the hops-distance estimates based on the differences in connectivity. The non-ranging positioning algorithm can be described as follows: Firstly, adopting the network to perceive information such as (ID, location, hops count, etc.) between the unknown node and the anchor node. Secondly, using information fusion and mathematical calculation methods to estimate the distance between nodes. Finally, the location of the unknown node can be estimated. The basic steps are:

(1) Using the protocol of distance vector exchange in sensor networks, the hop count *h* and distanced information between unknown nodes and anchor nodes are collected. In the network, packets containing location information are forwarded until all nodes are aware of the location of each anchor node. With data fusion technology, data in all packages is associated.
(2) According to the location information of other anchor nodes received by the known node, the hop-distance conversion model is established. Using the distance formula, we can estimate the actual distance about per hop, and then broadcast it over the entire network.
(3) We can obtain the estimated distance between the unknown target node and each anchor node. By applying the mathematical method (triangular method and maximum likelihood estimation method), we can further estimate the position coordinates of the target node and correct the calibration.

### 3.1. Mechanism of Data Broadcasting

**Figure 5:**
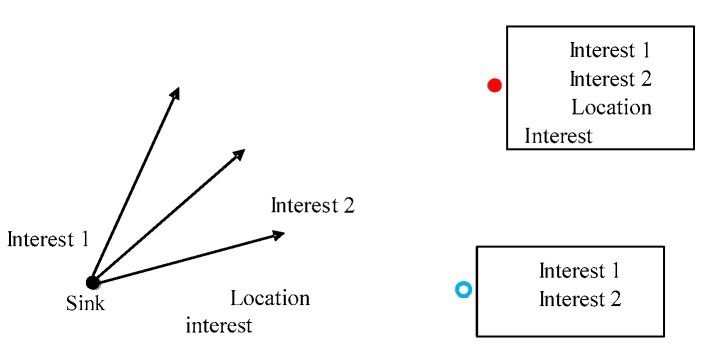
The broadcast flooding mechanism in network.

The maximum communication radius of an anchor node is the range of information transmission, and the typical network protocol of distance vector exchange is used to send packets to the surrounding neighbor nodes in a broadcast manner. This protocol uses bellman-ford to calculate the path. In the distance-vector routing protocol, each router does not know the topology information of the entire network. They simply announce their distance to the other routers and receive similar notices from them. The packet contains its own location coordinate information, the current time information and accumulated hops information. The packet format can be expressed as: LocInf[(*x_i_*,*y_i_*),*ID_i_*,*T*]. At this time, the location information is transmitted as a normal data message. The sink node initiates the “interest” for the detection target of the location. If an anchor node responds to this “interest” message, its own location information is immediately broadcast and forwarded as the collected data. As shown in Figure 5.

The random movement of nodes causes the data packet to generate multiple collisions during the broadcast, resulting in the loss of transmission information [33,34]. In order to avoid the above situation and protect the network performance, we set the maximum data transmission hop value *h_max_* in the limited broadcast range. As shown in Figure 5, the sink node does not save the “location information” message after the “interest” message is broadcasted. Only the source node with the anchor function records the “Location interest” message and returns the collected data. At this point, the data which is passed back is the information contained in the anchor node.

The special topology of the belt-type sensor networks determines that its internal nodes have different connectivity from other wireless networks. If we use this feature, we can directly identify the approximate orientation of the unknown node. A member node *S_k_* in the network issues a “Neighboring_request” message as soon as it finds another node which is in the same group. Within the effective communication range of the node, the message spreads quickly along the route. When the neighbor node of *S_k_* receives the message, it sends the reply message “Neighboring_act” at once. When its neighbor node receives the packet, it immediately checks the information in it, records the relevant information of the anchor node, and creates an anchor node to access the visitor list. In this process, the node is likely to receive multiple packets from the same anchor node. By checking and comparing, the nodes only retain the group of information which has the least number of hops in its grouping. Subsequently, the hop distances from each anchor node in the bounded area are accumulated and added “1” to their hop count. Lately the packet is forwarded to the surrounding node. The network repeats this process until all nodes in the sensor networks record their location coordinate of each anchor node and the information of corresponding cumulative hop count. Through the above mechanism, all the sensor nodes in the belt-type sensor networks system obtain the cumulative hop distance and the minimum hop count of each anchor node.

For any node that receives a message from an anchor node, we use “*h_max_*” as the restriction range parameter for the discrimination. After adding the maximum number of hops, the unknown node is restricted to the extent of the anchor node information. It reduces the range of errors that can occur during packet delivery. More importantly, the use of this parameter reduces the probability of collision with interference information. If the message from the corresponding anchor node has been recorded in the node and the hop count value “*k*” is less than or equal to the newly received message hops, the newly received message of the node may be considered as an invalid message. Once the node receives a message that is defined as invalid, it immediately discards it and does not proceed with further processing in subsequent stages.

### 3.2. Correcting the Accumulated Values 

In the framework of standard DV-Hop algorithm, the estimated distances of the target node to the anchor nodes in the range of the surrounding hop are very easy to identify as the same, which makes it difficult to determine the distances between the adjacent nodes. A large number of measured statistical results show that if the cumulative number of hops are used to estimate the distances between the target nodes and any anchor nodes, the estimated results are greater than the actual physical distance between them [21,35]. In the algorithm process, an appropriate correction method must be added in its framework when the estimation distances and coordinate calculation are carried out. In this way, it is possible to avoid the accumulation of errors between the anchor nodes and the positioning target node due to the accumulation of the positioning information. It also can reduce the accuracy of the positioning accuracy due to the blurring of the hops-distance estimation. Our purpose is to consider the differences between the distance between adjacent nodes (anchor nodes and neighbor nodes) and network connectivity. To improve the accuracy of positioning estimation is our ultimate goal. Based on the above reasons, an auxiliary distance correction estimation method based on the optimization of connectivity among nodes is proposed without adding additional communication overhead. Let the average hop distance of the network be expressed as *d_i_*, and its expression can be defined as:

(4) $$d_{i}=\frac{\sum_{i\neq j}md_{ij}}{\sum_{i\neq j}h_{ij}}$$

In Equation (4), *md_ij_* represents the cumulative hops distance between the anchor and the target, and *h_ij_* represents the minimum value of the direct hops between the two. According to the principle that in the network the communication radius of nodes is greater than the distance between nodes, we uniformly collect the average distance per hop of multiple recent anchors received by the target node, and then weighted processing of them is normalized. Firstly, $\theta_{m}$ is defined as the average distance error between nodes *m* and *n*.

(5) $$\theta_{m}=\frac{\sum_{m\neq n}\left|d_{est(m,n)}-d_{r(m,n)}\right|/h_{mn}}{M-1}$$

In Equation (5), $d_{est(m,n)}$ represents the estimated distance between nodes *m* and *n*. $d_{r(m,n)}$ represents the actual distance between them. Assuming that the target node receives the data sent by the *k* anchor nodes at this time, the average hop-distance weighting coefficient from the $m^{\mathrm{th}}$ anchor node is expressed as:

(6) $$\eta_{m}=\frac{1}{\theta_{m}}/\sum_{k=1}^{n}\frac{1}{\theta_{k}}$$

In summary, when the member nodes of the network receive the distance value represented by the average hop per hop (*h*) through broadcasting, the distance estimation to the corresponding anchor node is expressed as $d_{est(i,j)}$. It is shown in Equation (7):

(7) $$d_{est(i,j)}=\eta_{m}\sum_{i\neq j}h_{ij}\times d_{i}$$

According to the principle of simplicity, we only select the last three anchor node information, and $\lambda_{m}$ is normalized. In a network, a single node can send test packets(*S*) to determine the neighbor relationship. We define γ as the threshold for the packet rate, which needs to meet the condition 1/*S* ≤ *γ* ≤ 1. When node *i* receives the packet scent by node *j*, if it is determined that the following relationship exists, node *i* and *j* can be determined as neighbors. A significant increase in the reliability of the node’s neighbor distance makes it possible to have a unique relationship between the different unknown nodes and its recent anchor nodes. Based on it, we can eliminate some information from interfering nodes or false anchors. After removing the tampered beacon information and eliminating the interfering nodes, we can obtain the relative accurate node distance, so as to correctly compute the node location. The higher the accuracy of the neighbor node selected by the neighbor of the node, the closer the final position estimate is to the real. According to the following two conclusions [36]: (I) the greater the connectivity difference between nodes, the greater the distance between neighbors, and (II), the greater the distance between neighbor nodes, the closer the communication radius is to the other nodes. When quantifying the difference characteristics of connectivity between network nodes, we could achieve the summation of the absolute values of the connectivity differences. For the unknown node *I* (target node) and its neighbor anchor node *m* to be located, the value of connectivity difference between the two can be expressed as:

(8) $$C_{im}=\sum_{i\neq m}\left|h_{ij}-h_{mj}\right|.$$

In the Equation (8), *h_ij_* is defined as the hop count value of the shortest connection path between the fixed anchor *j* and the unknown node *i* which should be located; *h_mj_* is defined as the shortest hops between the neighbor anchor node *m* and the anchor node *j*.

Thus, the neighbor distance of the maximum difference between the target node and undetermined target node *i* can be set as the communication radius of the node ($R_{c}$). The ratio of the node connectivity difference value *C_im_* obtained by Equation (8) to the maximum difference value *C_iMAX_* in the network is taken as the weight value. We can multiply the ratio with the node communication radius to obtain the actual distance *d_im_* from the target node *i* to the $m^{\mathrm{th}}$ anchor node. *d_im_* is calculated as follows:

(9) $$d_{im}=\sum_{i\neq m}C_{im}\times \eta_{m}\times R_{c}/C_{iMAX}.$$

We improve the method of estimating the hop count of the whole communication link, correct the distance value represented by each hop between the target node and the anchor node. The above work can more accurately represent and reflect the true connection of nodes in sensor networks. These jobs lay the key foundation for further reduction of positioning errors.

### 3.3. Locating the Node and Fix the Result 

Once the target node obtains three or more distance parameters of the anchor nodes, the triangulation positioning method is run immediately. Let the coordinate of the anchor node be $\left(x_{n},y_{n}\right)$, its cumulative distance to the unknown node (target node) is $d_{n}$, and coordinate of the unknown node is (x,y). According to the distance relationship between two points, the distance system between the different anchor node and the target node is established as shown in Equation (10):

(10) $$\left\{ \begin{array}{c} {\left(x-x_{1}\right)}^{2}+{\left(y-y_{1}\right)}^{2}=d_{1}^{2}\\ {\left(x-x_{2}\right)}^{2}+{\left(y-y_{2}\right)}^{2}=d_{2}^{2}\\ \cdots \cdots \cdots \cdots \cdots \cdots \cdots \cdots \cdots \\ {\left(x-x_{n}\right)}^{2}+{\left(y-y_{n}\right)}^{2}=d_{n}^{2} \end{array}\right.$$

According to the difference of the connectivity of the neighboring nodes, if the hop count is used directly to estimate the distance, the result is bound to be erroneous. When adopting the trilateral method to locate nodes, the process will produce many circles with different radius lengths. In essence, these equations of the circles with different radius are difficult to converge precisely at one point, which may cause the system to produce no solution. We can adopt the least squares method to obtain a set of approximate solutions for the unknown node. The expression after conversion is given by Equation (11):

(11) AX=b

The matrix forms of *A* and *b* are shown in Equations (12) and (13):

(12) $$A=\left[\begin{array}{ccc} 2(x_{1}-x_{n}) & & 2(y_{1}-y_{n})\\ \vdots & & \vdots \\ 2(x_{n-1}-x_{n}) & & 2(y_{n-1}-y_{n}) \end{array}\right]$$

(13) $$b=\left[\begin{array}{l} x_{1}^{2}-x_{n}^{2}+y_{1}^{2}-y_{n}^{2}+d_{kn}^{2}-d_{k1}^{2}\\ \vdots \\ x_{n-1}^{2}-x_{n}^{2}+y_{n-1}^{2}-y_{n}^{2}+d_{kn}^{2}-d_{k(n-1)}^{2} \end{array}\right]$$

The matrix form of the unknown node coordinates to be positioned is Equation (14):

(14) $$X=\left[\begin{array}{l} x\\ y \end{array}\right]$$

According to the estimation method of the standard minimum mean square error, we can calculate the following result:

(15) $$\hat{x}={\left(A^{T}A\right)}^{-1}A^{T}b$$

In order to improve the distance estimation between nodes, to maximize the credibility of the distance between the neighbor anchor nodes and to improve the positioning accuracy of the ribbon network based on connectivity, the estimated position of the unknown target node needs to coordinate the calibration. The purpose of doing that is to make the estimated position constantly approach the true position. The algorithm can introduce a special mathematical control method according to the distance constraint relation between neighbor nodes with high reliability weights. It can improve the accuracy of the location results by making finite iteration updates to the subsequent node coordinates after the three-side measurement. If we adopt the recursive form of a Taylor series expansion, we need to use the results which have been achieved the last time as an initial value. The local solution of the measurement error is obtained by the least squares method, and the iteration value of the operation is obtained. The algorithm needs to fix the position of the node constantly, and the operation ends when the error reaches the pre-set threshold. The Taylor series expansion method is suitable for the positioning systems with three or more base stations. It is necessary to ensure that the process is convergent. However, this method is more dependent on the selected initial value. Also, the computational complexity is higher and cannot run in a single node. In this paper, we use the gradient descent algorithm which is relatively simple to correct the estimation results. This method can improve the positioning accuracy by optimizing and improving the target position estimation.

The gradient descent algorithm is a minimized optimization method based on the Newton principle [8]. According to the properties of the proposed Newtonian iteration, the magnitude of the gradient represents an approximation of an estimate and an optimal point. The smaller the gradient, the closer the estimate is to the best position. When the result of the algorithm converges to the least square solution, the normal anchor node will continue to search along the normal gradient direction rapidly, while the false anchor node will deviate from this gradient direction. This method can also eliminate some of the anchors that are interrupted or maliciously tampered with. The core principle of this algorithm is: to set up the equivalent of the equations to convert, to build a multivariate function of the minimum value for the problem, and then solve it. In terms of convergence speed, the convergence rate is faster when its initial value is far from the true value, and then approaches the initial value. Newton method convergence can basically meet the requirements of connectivity-based positioning system. However, when using it we must obtain the partial derivative of each function. In that way, it is not conducive to rapid solution, or meets the reality that the computing power of the sensor node is weak. In contrast, the computational process of the gradient descent algorithm is relatively simple and does not require high precision of the initial point. It can quickly adjust the initial value to the real value while ensuring the convergence of the algorithm. More importantly, it is faster than the overall iteration. So this method is very suitable for fast operation in a single node. Thus, according to its principle, we can construct a system of the equations representing the distance between two points as a modular function. First, it is rewritten as Equation (16) shown in the equivalent form:

(16) $$\left\{ \begin{array}{l} f_{1}{}_{}(x,y,D_{k1})=0\\ f_{n}(x,y,D_{kn})=0 \end{array}\right.$$

In that case, the modulo function can be used to optimize the position estimation. Its expression is shown in Equation (17):

(17) $$f_{i}(x,y,D_{ki})=\sqrt{{\left(x-x_{i}\right)}^{2}+{\left(y-y_{i}\right)}^{2}}-D_{ki}$$

At this point, the question is how to get the minimum value of Equation (17). According to the Equation (14), we construct an optimized modular function expression. As shown in Equation (18):

(18) $$F(x,y,D_{k1},D_{k2},\cdots ,D_{kn})=\sum_{i=1}^{n}f_{i}^{2}(x,y,D_{ki})$$

The zero-point of the Equation (18) is obtained as the solution of the Equation (17). According to that principle, the function $f_{i}^{2}(x,y,D_{ki})$ can be regarded as a spatial surface in geometry, and the point tangent to the coordinate plane is the zero minimum point. For any point within the domain of the function definition, there is always a contour line passing through it. Starting from the point $\left(x_{0},y_{0}\right)$, moving in the direction of the monotonic decline of the function, the solution to the problem can be obtained by reaching the zero minimum. According to the plane geometry definition, the gradient direction at a point is the normal of the contour of the point. Therefore, its negative direction is the fastest direction of the decline of functions. Specific steps are as follows:

Step 1: Algorithm Initialization: calculate the initial value, select the optimal step size, and find the gradient value. We assign values to $\left(x_{{}^{\left(0\right)}},y_{{}^{\left(0\right)}}\right)$ and introduce parameters λ to get a new point $\left(x_{{}^{\left(1\right)}},y_{{}^{\left(1\right)}}\right)$:

(19) $$\left\{ \begin{array}{l} x_{{}^{\left(1\right)}}=x_{{}^{\left(0\right)}}-\lambda g\\ y_{{}^{\left(1\right)}}=y_{{}^{\left(0\right)}}-\lambda g \end{array}\right.$$

In Equation (19), $x_{{}^{\left(0\right)}},y_{{}^{\left(0\right)}}$ can be calculated by least squares method. By using Equation (9), we can get $d_{kn}{}_{{}^{\left(0\right)}}$.

Step 2: We find λ and put $\left(x_{{}^{\left(1\right)}},y_{{}^{\left(1\right)}},d_{k1}{}_{{}^{\left(1\right)}},d_{k2}{}_{{}^{\left(1\right)}},\cdots ,d_{kn}{}_{{}^{\left(1\right)}}\right)$ into Equation (20) in the iterative operation, until the unknown value is equal to $\left(x_{{}^{\left(k\right)}},y_{{}^{\left(k\right)}},d_{k1}{}_{{}^{\left(k\right)}},d_{k2}{}_{{}^{\left(k\right)}},\cdots ,d_{kn}{}_{{}^{\left(k\right)}}\right)$. The mode function $F_{k}$ is transformed into Equation (20):

(20) $$F_{k}=F(x_{{}^{\left(k\right)}},y_{{}^{\left(k\right)}},d_{k1}{}_{{}^{\left(k\right)}},d_{k2}{}_{{}^{\left(k\right)}},\cdots ,d_{kn}{}_{{}^{\left(k\right)}})$$

Step 3: When |F| is less than the set permission error, the $\left(x_{{}^{\left(k\right)}},y_{{}^{\left(k\right)}},d_{k1}{}_{{}^{\left(k\right)}},d_{k2}{}_{{}^{\left(k\right)}},\cdots ,d_{kn}{}_{{}^{\left(k\right)}}\right)$ is the solution, the algorithm process ends. Otherwise, go back to Step 4 for processing again.

Step 4: According to Equation (21), the difference quotient $\left(F_{{}^{\left(k\right)}}/x,F_{{}^{\left(k\right)}}/y\right)$ is calculated:

(21) $$\begin{array}{l} F_{{}^{\left(k\right)}}/x=F(x_{{}^{\left(k\right)}}+x,y_{{}^{\left(k\right)}},d_{k1}{}_{{}^{\left(k\right)}},d_{k2}{}_{{}^{\left(k\right)}},\cdots ,d_{kn}{}_{{}^{\left(k\right)}})/x\\ -F(x_{{}^{\left(k\right)}},y_{{}^{\left(k\right)}},d_{k1}{}_{{}^{\left(k\right)}},d_{k2}{}_{{}^{\left(k\right)}},\cdots ,d_{kn}{}_{{}^{\left(k\right)}})/x\\ F_{{}^{\left(k\right)}}/y=F(x_{{}^{\left(k\right)}}+x,y_{{}^{\left(k\right)}},d_{k1}{}_{{}^{\left(k\right)}},d_{k2}{}_{{}^{\left(k\right)}},\cdots ,d_{kn}{}_{{}^{\left(k\right)}})/y\\ -F(x_{{}^{\left(k\right)}},y_{{}^{\left(k\right)}},d_{k1}{}_{{}^{\left(k\right)}},d_{k2}{}_{{}^{\left(k\right)}},\cdots ,d_{kn}{}_{{}^{\left(k\right)}})/y \end{array}$$

Step 5: We calculate the one-step predictor, and then Equations (22) and (23) are established:

(22) $$x_{{}^{\left(k+1\right)}}=x_{{}^{k}}-W_{{}^{k}}{\left[F/x\right]}_{{}^{k}}$$

(23) $$y_{{}^{\left(k+1\right)}}=y_{{}^{k}}-W_{{}^{k}}{\left[F/y\right]}_{{}^{k}}$$

In Equations (22) and (23), the following relationship exists, as shown in Equation (24):

(24) $$W_{{}^{k}}=\frac{F(x_{{}^{\left(k\right)}},y_{{}^{\left(k\right)}},d_{k1}{}_{{}^{\left(k\right)}},d_{k2}{}_{{}^{\left(k\right)}},\cdots ,d_{kn}{}_{{}^{\left(k\right)}})}{{\left[F_{\left(k\right)}/x\right]}^{2}+{\left[F_{\left(k\right)}/y\right]}^{2}}$$

Step 6: According to the need for positioning accuracy, jump to Step 2 again, the iterative cycle calculation. Otherwise, the algorithm should be terminated.

While adopting the gradient descent algorithm in the Gaussian channel environment, we should select and search the optimal solution in the controllable range on the basis of the initial value [21]. At the same time, for the whole positioning process, we use the results of the least squares algorithm to provide more accurate initial information for gradient descent algorithm. More importantly, it has not been necessary to add additional communication overhead to obtain the other conditions required for the calculation, which is very useful for belt-type topologies with limited computing power.

## 4. Simulation and Analysis

**Figure 6:**
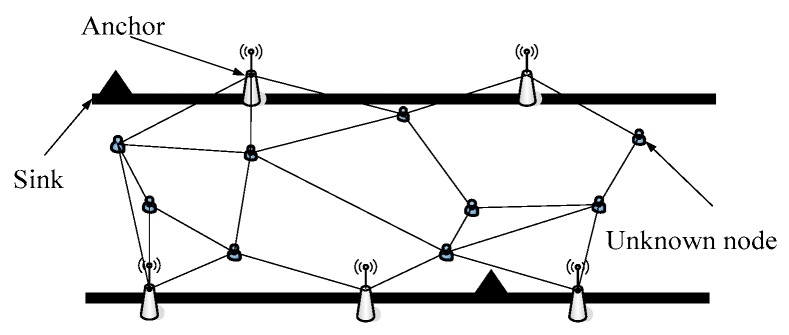
Process of target locating in belt-type sensor networks.

In this section, we simulate and analyze the performance of the proposed algorithm. In order to verify the effectiveness of the improved algorithm, we use Matlab for validation. In accordance with the height of the real scenario, we set the network environment similar to the narrow strip area (shown in Figure 6).

The sensor nodes are deployed in the network, the horizontal spacing distance *L* is set to 1000 m, and the longitudinal distance *M* is set to 15 m. The anchor nodes are arranged along the edge of the runway according to the strategy described in Section 3.3, and the target nodes to be positioned are randomly arranged in the network coverage area. The overall network layout is shown in Figure 6. In this paper, the standard DV-Hop algorithm, DV-Distance algorithm and improved CDV-Hop algorithm are introduced in our experiment. 

### 4.1. Relative Error of Positioning Accuracy

*Error_ave_* is the ratio of the target positioning error to the communication radius of the node itself. It is defined as the relative error of positioning accuracy and its expression is (25):

(25) $$\begin{array}{l} Error_{ave}=\frac{\sum_{i=1}^{n}\Delta r_{i}}{N\times R_{c}}\times 100\%\\ =\frac{\sum_{i=1}^{n}\sqrt{{\left(x_{i,est}-x_{i,real}\right)}^{2}+{\left(y_{i,est}-y_{i,real}\right)}^{2}}}{N\times R_{c}} \end{array}$$

In Equation (25), the coordinates of the target node are denoted as $\left(x_{i,est},y_{i,est}\right)$, the coordinates of the real position of the target node are denoted by $\left(x_{i,real},y_{i,real}\right)$, *N* is the number of unknown target nodes, and $R_{t}$ is the communication radius of the normal node.

**Figure 7:**
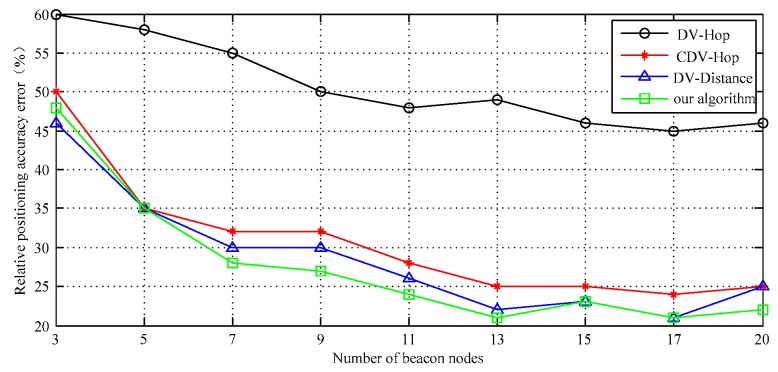
Localization error vs. Number of anchors.

It can be seen from Figure 7 that the error of the average positioning accuracy for the target is maintained at 27% when the number of anchor nodes is about 11 and the other network conditions are unchanged. If the number of anchor nodes increases from 3 to 13, the algorithm positioning error basically displays decreasing trend. The standard DV-Hop has the largest positioning error because it does not use other corrective measures. However, when the number of anchor nodes reaches 13 or 14, the trend of positioning accuracy error is almost smooth. As the band network continues to extend along the edge, the difference between adjacent anchor nodes is narrowing, and their correction to distance estimation is weakening.

In addition, when the anchor nodes increase to a certain percentage, the network connectivity no longer significantly changes. Therefore, when the number of anchor nodes reaches 13 or so, the positioning error basically becomes stable. When the number of anchor nodes is further increased, the positioning accuracy of the DV-Distance algorithm is slightly decreased. That is because the DV-Distance algorithm must use the RSSI signal as an auxiliary estimation parameter. When the signal transmission is within a certain distance range, its amplitude change, resulting in increased signal fluctuations. The number of anchor nodes can increase the source of information needed for locating. But on the other hand, it also introduces more error sources to some extent. When such a node reaches a certain number, it can lead to an increase in cumulative errors and relatively negative effects on the elevation of positioning accuracy.

**Figure 8:**
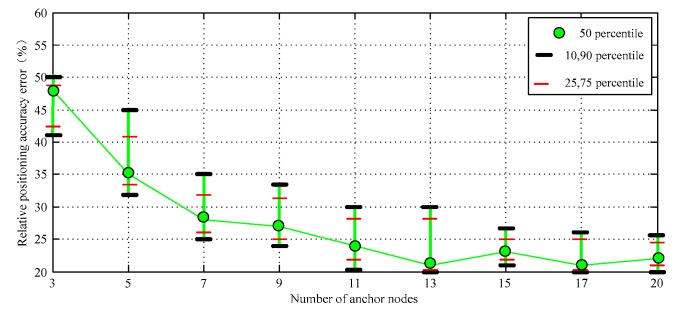
The confidence interval of the data I.

The simulation data curve in Figure 7 shows that the relative positioning accuracy error of DV-Distance and proposed algorithm is significantly lower in general. When the number of nodes is less than 13, the precision of the proposed algorithm is slightly higher than that of DV-Distance algorithm. According to the simulation process, the proposed algorithm only needs to be carried out two iterations, the initial value is approaching the real value quickly. It shows some advantages: the convergence speed is faster, the computation is small. The confidence interval of the simulation results is shown in Figure 8.

**Figure 9:**
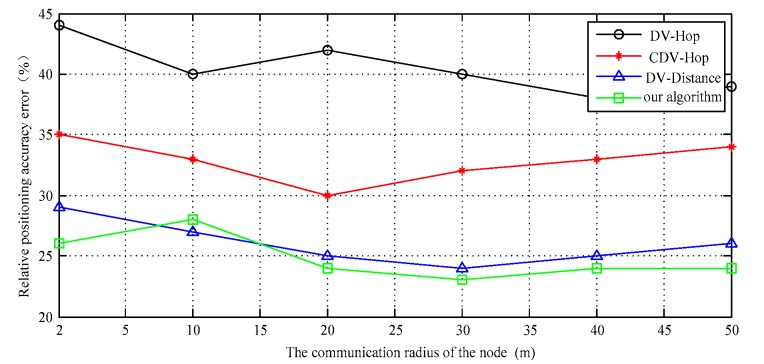
Localization error vs. Node communication radius.

It can be seen from Figure 9, from the perspective of qualitative analysis, that the communication radius of a node can determine the number of its neighbors and number of connections. As the communication radius of the target node increases, it can be searched and acquired the location information for more anchor nodes within its communication range.

**Figure 10:**
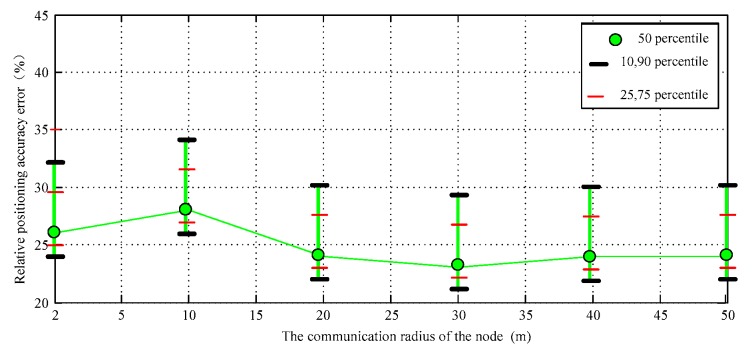
The confidence interval of the data II.

At the same time, the radius of node communication increases, which can also make its own positioning coverage increase. In that way, the node can obtain more adjacent node location information, so the estimation accuracy can be improved. DV-Distance algorithm uses RSSI signal as auxiliary positioning parameters. If the distance between nodes is more than 20 m, the signal fading curve will show large fluctuations. At this moment, the communication radius can no longer directly improve the positioning accuracy, the overall positioning accuracy and the proposed improvement algorithm is basically close, but it uses additional hardware that can measure distance which greatly increases the communication consumption and reduces efficiency, so the positioning cost is also affected. The confidence interval of the simulation results is shown in Figure 10.

**Figure 11:**
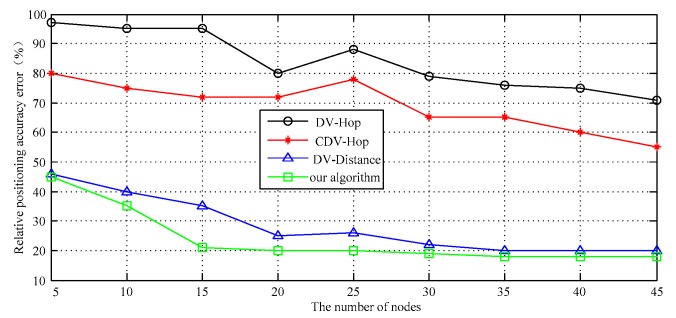
Localization error vs. Total number of sensor nodes.

**Figure 12:**
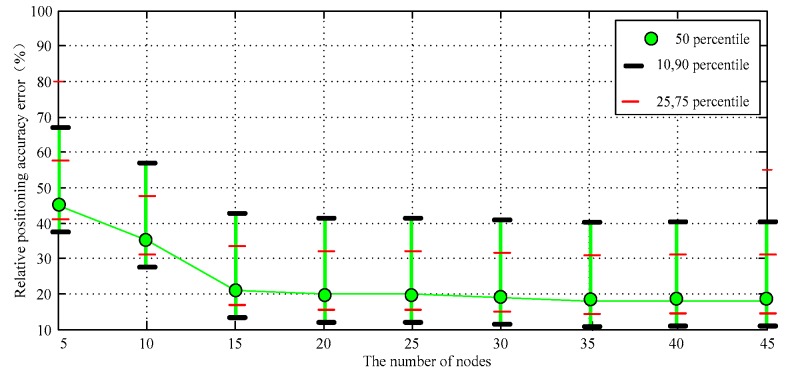
The confidence interval of the data III.

At a certain time, we gradually increase the number of unknown randomly distributed target nodes. The relationship between node number and location accuracy is shown in Figure 11. We see that the error of proposed algorithm is significantly smaller than the DV-Distance algorithm, the DV-Hop algorithm and CDV-Hop algorithm. On the whole, the larger number of nodes is beneficial to improve the accuracy of the distance estimation between nodes. That is because the localization algorithm using network connectivity (hop number) is designed based on the relationship between the number of hops and distances between nodes. The network connectivity will directly influence the accuracy of the algorithm. The confidence interval of the simulation results is shown in Figure 12.

Network connectivity affects the accuracy of the algorithm directly. The better the network connectivity, the more accurate the distance between each hop. Connectivity refers to the existence of at least one direct path between any two nodes in the network, enabling efficient communication [16]. According to the basic theory of graph theory [26,27], the minimum node degree of the network is expressed as $D_{\min}(G)$:

(26) $$D_{\min}(G)=\min\left\{D(v)\right\}$$

In Equation (26): v represents a node in the network, and *D*(*ν*) represents the number of neighbor nodes that can communicate with the node directly. In the network the connectivity of any node with node *N* and the remaining *N* − 1 nodes can be expressed as K, and the relationship can be expressed as (27):

(27) $$E(K)=(N-1)p_{x}$$

In Equation (27): $p_{x}$ is the probability of any two nodes being connected when the communication distance is fixed. The increase in connectivity index $p_{x}$ indicates that the number of hops between nodes decreases, and the average positioning error decreases with the increase of node density. The reason is that although the node position is relatively dispersed, its probability density function is continuous. When the node density increases, the discrete function approximates the continuous function. This can make the error smaller, thus improving the positioning accuracy. The better the connectivity, the closer to the true value for each hop estimate. When calculating the coordinates, the gradient method can be used to make the evaluation more accurate, so the estimation accuracy is higher. However, due to the number of anchor nodes has not changed, other nodes lead to an increase in its proportion, the whole error curve is not always showing a downward trend.

### 4.2. The Time Required to Locate Nodes

To a certain extent, the time required to complete the positioning can reflect the complexity of the positioning algorithm, and reflect how much energy is generated in the positioning process indirectly. The relationship between the number of anchor nodes and the time required for positioning is shown in Table 1, it can be seen that the anchor nodes have a relatively large number of tasks, including collecting hops information and collating and forwarding packets, so the direct impact on time is most obvious. Compared with the standard DV-Hop, DV-Distance increases the process of calculating the signal attenuation value. CDV-Hop increases the coordinate calibration after estimating, the improved algorithm adds the correction and iterative refinement process in the distance estimation and trilateral positioning, so the computational complexity of the three algorithms is increased, and the time required is relatively long. The CDV-Hop and DV-Hop algorithms are relatively simple, so the computational complexity is low, and they can be applied to those occasions that do not require high precision.

Under normal circumstances, it can be seen from Table 1 that the time required for positioning will increase as the number of nodes participating in the network increases. The increasing number of network nodes means that the consumption time of information transmission and data acquisition also increases correspondingly. At the same time, the increase of data volume also increases the time required to calculate.

When the topology of the wireless sensor network presents a long band, the connectivity of the nodes is changed to a certain extent, and the time required for different algorithms will be different. The number of nodes in the network and the time required for positioning are shown in Table 2. It can be seen that when the number of nodes participating in the positioning increases from 10 to 50, the range of DV-Distance and proposed algorithm is less than 500 ms~600 ms, while DV-Hop and CDV-Hop are nearly doubled. 

## 5. Conclusions

Reliable and secure positioning algorithm is a significant issue in the IoT. In this work, we focus on the problem of target location in belt-type sensor networks. An energy-efficient strategy is adopted to deploy the IoT nodes, and decision mechanism of the broadcasting data is utilized to improve the security and reliability of the positioning information. We apply a new method to estimate the hop distances by relating the proximity of the neighbors to their connectivity difference. By weighting the average hop distance error of the anchor nodes, the average hop distance is modified and more accurate neighborhood distances are calculated. Through the improved strategy, compared with previous methods, the proposed algorithm effectively enhances the speed and precision of the positioning, reduces the possibility of the anchor nodes suffering abnormal interference, and meet the needs of energy efficiency. The main factors influencing the localization errors are analyzed by simulation, and the validity of the algorithm is verified as well. Theoretical analysis and validation results shows that our location algorithm has a good effect in detecting false anchor nodes and resisting interference attacks. Compared with current non-ranging methods, our algorithm improves the positioning accuracy by nearly 19%. The results also reveal that the proposed localization algorithm is more convenient. It has demonstrated some good characteristics in fast convergence, extension performance and computation amount. With the development of wireless technology, the value of the belt-type sensor networks will be further recognized and utilized in next generation IoT systems. Our next step will continue to focus on improving the accuracy, security, and energy-efficient of positioning. More deployment experiments will be executed.

## Figures and Tables

**Table 1:** Effect factors of localization time (case 1).

Algorithm Type	The Number of Anchor Nodes			
	3	7	10	15
DV-Hop	200 ms	210 ms	270 ms	310 ms
C DV-Hop	400 ms	420 ms	550 ms	590 ms
DV-Distance	1350 ms	1500 ms	1820 ms	1970 ms
Our algorithm	1200 ms	1200 ms	1500 ms	1550 ms

**Table 2:** Effect factors of localization time (case 2).

Algorithm Type	The Number of Sensor Nodes			
	10	20	30	50
DV-Hop	1200 ms	1200 ms	1500 ms	1500 ms
C DV-Hop	900 ms	1000 ms	1600 ms	1750 ms
DV-Distance	1400 ms	1500 ms	1700 ms	17,500 ms
Our algorithm	1700 ms	1750 ms	1900 ms	1950 ms
